# A Treatise on Orthopedic Surgery

**Published:** 1910-12

**Authors:** 


**Difficult Labor.** By **G. Ernest Herman,** M. B. Lond., Consulting Obstetric Physician to the London Hospital. 12 mo. pp. 559. Illustrated. New York: William Wood & Co., 1910. (Cloth, $2.50.)

The fifth edition of Herman's book follows along the lines adopted in previous revisions. His advice as to the management of difficult cases of labor is unusually sound and conservative. A chapter on puerperal eclampsia has been added to the new book in which the author advocates what he terms the morphia treatment. The summary of his treatment for eclampsia is quoted: "You are called to a patient who has had one or more fits; her urine is scanty and when boiled solid with albumin; she is insensible but restless. Give ½ gr. morphia with 1-60 gr. atropine by hypodermic injection. In half an hour, if the patient is not in deep sleep repeat the dose. If in half an hour after that the patient is still restless give ¼ gr. of morphia with 1-120 grain of atropine, and repeat every half hour until the patient is in a deep sleep."

---

**Transactions of the American Dermatological Association** at its thirty-third annual meeting held in Philadelphia, June 3-5, 1909. **Grover W. Wende,** M.D., secretary.

The meeting, of which this volume chronicles the proceedings was held at Philadelphia, June 3, 4 and 5, 1909, under the presidency of Dr. Thomas Caspar Gilchrist, of Baltimore. The book shows careful preparation and editing by the secretary, Dr. Grover William Wende, of Buffalo. One excellent feature of the transactions is that a list of publications of the members for the year preceding the meeting is printed at the end. A fine group of papers is published, most of them on technical subjects, and many of them are well illustrated. Dr. Wende, himself, publishes an article entitled "A Nodular Terminating in a Ring Erruption of the Skin," otherwise granuloma annulare. This interesting case is described in detail and a graphic illustration of the patient is published. The volume is of great interest to every one who practises dermatology.

---

**A Treatise on Orthopedic Surgery.** By **Royal Whitman,** M.D., Adjunct Professor of Orthopedic Surgery in the College of Physicians and Surgeons, New York; Professor of Orthopedic Surgery in the New York Polyclinic. Fourth edition. Octavo, 908 pages, with 601 illustrations mostly original. Lea & Febiger, Publishers, Philadelphia and New York. 1910. (Cloth, $5.50, net.)

Whitman's treatise for years has stood for all that is best in orthopedic surgery, and this recently revised edition establishes more firmly than before the high position held by its predecessors. The author gives us the result of his own extensive experience and has brought before us a very complete view of the theory and practice of this branch of surgery. The opportunity to prevent life-long deformity by correction applied when the body is young and plastic brings the subject within the sphere of the family practitioner, for it is he who first sees such defects. They form also a large share of general surgery, hence the work will be of interest to the surgeon and specialist. The entire book has been reset. Many new illustrations appear. The author has added statistical and other data that will be of interest to workers in this special field. J. A. R.

